# Aquatic Physical Literacy: The Effectiveness of Applied Pedagogy on Parents’ and Children’s Perceptions of Aquatic Motor Competence

**DOI:** 10.3390/ijerph182010847

**Published:** 2021-10-15

**Authors:** Pietro Luigi Invernizzi, Marta Rigon, Gabriele Signorini, Giampiero Alberti, Gaetano Raiola, Andrea Bosio

**Affiliations:** 1 Department of Biomedical Sciences for Health, Università degli Studi di Milano, 20129 Milan, Italy; pietro.invernizzi1@unimi.it (P.L.I.); marta.rigon@unimi.it (M.R.); gabriele.signorini@unimi.it (G.S.); giampietro.alberti@unimi.it (G.A.); 2 Department of Human, Philosophical and Education Sciences, University of Salerno, 84084 Fisciano, Italy; graiola@unisa.it; 3 Human Performance Laboratory, Mapei Sport Research Centre, 21057 Olgiate Olona, Italy

**Keywords:** grounded theory, swimming, linear pedagogy, non-linear pedagogy

## Abstract

The goal of swimming school during early school age is to promote physical literacy. According to linear or non-linear pedagogy, a linear or non-linear approach teaching style can be used. The aim of our study was to investigate whether a different teaching methodology, as in using a teacher-centered approach (linear pedagogy), or a student-centered approach (non-linear pedagogy), could differently influence the perceptions of aquatic activity of children and parents. Parents of 100 children (53 females and 47 males, age 5.9 ± 0.3 years old) participating in the study were previously interviewed to ascertain their expectations regarding the swimming course. Participants were in a medium-high social context. Subsequently, considering the data of the incoming aquatic motor competence’s tests, children were divided into a linear (LI) and non-linear (NL) pedagogy group. A total of 4 instructors were enrolled. Upon completing the swimming course, the aquatic motor competence’s test was repeated, a pictorial scale of perceived motor competence was administered, and a questionnaire regarding the course was proposed to children’s parents. Physical development and learn to swim resulted two of the most important reasons leading parents to choose swimming courses. LI achieved greater progress than the NL in actual motor competence (A.M.C.), while NL perceived a higher aquatic motor competence (P.M.C.) in 7 out of 10 items of the pictorial scale. Parents of children in LI, gave greater importance to the teacher’s role, while NL’s parents pointed the acquisition of children’s abilities as pivotal. In conclusion, NL approach was more appreciated by children, while LI method was more rewarding for parents because initial expectations were satisfied.

## 1. Introduction

The acquisition of good health practices, the awareness of the benefits of physical activity and the acquisition of motor skills are the basis for the development of appropriate motor literature leading to the maintenance of healthy behavior throughout life. Physical literacy can be described as the potential which everyone has in terms of motivation, confidence, physical competence, knowledge and understanding to assess and assume the responsibility for maintaining goals of physical activity throughout their lives [1].

Whether this potential is expressed or not depends on the motor competence, the actual physical activity carried out and the cultural context [1]. In this regard, each child needs to perform an adequate quantity of physical activity level in order to achieve the proficiency barrier, related to the adequate physical activity levels for health [2].

Water physical activity is a means for cognitive and motor development: practical and senso-motor intelligence constitutes the basis for the development of verbal and cognitive intelligence. It can be asserted that there’s a continuity between intelligence and biological process of adaptations to the environment [3,4]. 

Through water practice, neuropsychic development of language [5] and children’s personality are promoted [6,7], which can be used in the socio-relational sphere. 

An early approach to aquatic environment allows an individual to become related to the outside environment with greater autonomy, decision-making independence and willingness. These skills enable the child to develop more self-confidence and promote the overcoming of fear, which could hinder the possibility to face new and unknown situations calmly [6]. 

In addition to psychosocial and cognitive development, swimming allows the development of strength, balance, flexibility, during the pre-scholar age [8], and the improvement of cardiovascular adaptation, leading to greater heart efficiency [9]. Swimming is therefore a useful skill for life and can promote the health status of an individual from a very young age.

The basis for learning aquatic motor competence are the development of movement, breathing and balancing techniques; altogether they constitute the bases for the safeness and comfort of an individual in the aquatic environment [10].

Balance in water is a skill to be acquired early because learning roll and body alignment, which promote good shoulder-shaped balance and reduce water resistance during moving, are more complex to internalize in adulthood [8].

Respecting the stages in learning swimming skills is very important: children until 5-years-old need a long period of psychological adaptation to the aquatic environment before being able to move with confidence and acquire specific skills [11]. Interaction with peers and the teacher will allow the development of languages and social skills as they become more self-confident. According to the social constructivism theory [12,13], cooperative learning in small groups facilitates the learning through the observation of the peers. Likewise, swimming instructor’s social competences are also fundamental to establish a relationship within the group [14]. Social competences could stimulate both child attention and curiosity, through an active involvement, and identify what the child already knows and what it is necessary that she/he learns. This process requires the teacher’s observation [15] and the consequent supply of appropriate feedback to consolidate what has been previously learned.

In this context the teacher’s role is more or less evident depending on the methodological approach that is used: “It is not sure which is the best teaching method, but it is clear that the most efficient program is one that respect each characteristic of the child, which are unique” [16]. The goal of the swimming school during early school age is to promote physical literacy [1]. In this respect, the choice of the teaching style to adopt could be directed towards a linear approach (linear pedagogy) [17]. With this method the instructor-facilitator proposes different activities based on imitative approach. This criterion is peculiar of reproductive styles, in which are determined a transfer of information as in the cognitivist approach [18], through the active interaction and the collaboration with the learner. Alternatively the approach can also be non-linear (non-linear pedagogy) [17]. It is an ecological-dynamic method based on productive styles in which the facilitator prepares the material in order to create new situations and intervenes when is occurred, without providing instructions to the group. Motor creativity and students’ autonomy are stimulated by the environment [19]. This allows the child to express himself freely and at the same time to address her/him towards a new motor representation of her/his body related to an environment which requires an important adaptation to the terrestrial motricity.

Even though there is no one teaching style better than another [16], we find it interesting to consider and investigate parents’ and children’s perceptions about their acquired competences through the use of the two teaching styles. Our researching field regards the scope of physical literacy and personal autonomy, the relationship between child and aquatic environment and the impact of the figure of the instructor both from a psychosocial and an organizational point of view. Starting from the survey concerning to the aquatic motor culture of the parent, the aim of our study was to investigate whether a different teaching methodology may influence, in a different way, the perceptions of aquatic activity of children and parents.

## 2. Materials and Methods

### 2.1. Participants and Context

A total of 100 children (age 5.9 ± 0.3 years old), 53 females and 47 males of a medium-high social context, with their parents, participated to the study for fifteen weeks, by organizing the activity into two weekly lessons of 50 min duration each. A total of four instructors were involved in the study, two for the linear pedagogy group (LI) and two for the non-linear pedagogy group (NL). During lessons instructor/children ratio was 1/8.

Eight children were excluded from the study due to their incomplete enrolment in the course, 5 for LI group and 3 for NL group.

To be included in the trial, children had to be 5–6 years old. Conversely, having previous swimming experience or having cognitive or motor disorders were considered as exclusion criteria.

An informed consent was signed by the parents before participating in the study and the trial was approved by the Ethics Committee of the University of Milan (protocol code 2/12).

### 2.2. Procedure

The parents of the children who participated in the study were interviewed in advance with the aim to express their expectations regarding the swimming course.

After the interview, children were divided into two groups considering the data of the incoming aquatic motor tests in order to obtain two groups with equal aquatic motor competence.

Linear pedagogy group (LI) was composed of 50 children, 23 females and 27 males (height 116 ± 6 cm; weight 22.7 ± 4.1 kg; Body Mass Index (BMI) 16.8 ± 2.3 kg/m²), the non-linear (NL) one was composed by 50 children, 30 females and 20 males (height 116 ± 6 cm; weight 21.4 ± 3.5; BMI 15.9 ± 1.4 kg/m²).

The groups were homogeneous at the baseline for the value of Langerdofer–Bruya [15] actual motor competence test.

LI group and NL (Table 1) group were involved in a period of swimming learning using linear and non-linear teaching styles, respectively, and at the end they were subjected to the aquatic motor tests again to verify which group had more benefits concerning actual motor competence. The two groups performed the lessons in pools with similar characteristics (shallow water; water temperature = 30 °C; environment temperature = 25 °C). Subsequently, the parents of the children in both groups responded to a multiple-choice questionnaire in order to verify the teaching style influence in parent’s perception of the swimming course. To evaluate the children’s self-perception at the end of their swimming course, children completed the pictorial scale of perceived aquatic competence.

### 2.3. Information Collection

#### 2.3.1. Preliminary Interview on Swimming Course Perception through the Grounded Theory (GT)

A preliminary interview was given to the parents of the children participating in the trial and was conducted according to the Grounded Theory’s model [22]. This approach involves a semi-structured interview that starts from the research questions and develops through the answers and argumentation of the interviewees. Therefore, no pre-defined questions were provided, but rather a general initial question from which different questions were developed according to the answers given by the respondents. The purpose of the interview was to investigate the aquatic motor culture of the parents. After this first phase, the coding of the answers was shared. In the first step of the opened coding, the themes called as pre-labels were extrapolated, those which expressed similar concepts were grouped in single theme defined labels. After the opened coding, the axial coding was done, dividing labels into categories, and quantifying them according to how often they appeared in the interviews. The labels appearing with a frequency less than 50% were defined as sporadic, the labels appearing with a frequency between 51% and 70% were defined as typical and the ones appearing with a frequency more than 71% were defined as general. From these, the Core Category was extrapolated, i.e., the emergent category. The answers were grouped regardless the child belong to the LI or NL group, and before the assignment to either group.

#### 2.3.2. Aquatic Motor Competence

Children were involved in aquatic motor tests to identify their initial level of actual motor competence. The same tests were repeated at the end of the course with the aim to verify the progresses achieved after the intervention. In this regard, the Langerdofer-Bruya [15] test was used, in order to investigate the water entrance capacity, the immersion, the breathing, the buoyancy, the movement of the arms, the recovery of the arms, the movement of the legs and the arms-legs coordination. The entrance in water with different orientations and the body’s inclination in water were not considered. The test was proposed by two neutral experienced researchers in this field, authors of this study, and not directly involved in the teaching.

#### 2.3.3. Parents’ Questionnaire (PQ)

After the intervention with the two teaching styles, a questionnaire with 12 multiple-choice questions was proposed to the parents, in which they had to give his/her opinion regarding the swimming course perception. The questions were structured on the basis of the answers obtained in the first interview with the grounded theory method and related to the three thematic areas emerged: physical literacy (PHY; 3 questions), child-aquatic environment relationship (CHI, 4 questions) and instructor (TEA, 5 questions). Physical literacy area regards what the swimming school teaches to the children in terms of wellness, confidence and specific competence; the children area refers to which aquatic competence the child achieves and learns from the course; the teacher area encloses all the aspects which can influence or promote through the teaching style adopted. Each question presented 4 possible answers, of which there were 2 negatives and 2 positives. Finally, the answers regarding positives perceptions of the parents were grouped and compared.

#### 2.3.4. Pictorial Scale of Perceived Aquatic Motor Competence (PS)

After the intervention with the two teaching styles, a pictorial scale of perceived aquatic motor competence was proposed. The questionnaire designed by Murcia and Ruiz Perez [23] is composed by 10 items in which it is possible providing an answer between the three choices and each option has a referred image in order to be easily understood by the children [23]. The questions referred to different scopes of aquatic motor competence also grouped in three thematic areas as the relationship with the swimming pool environment (related to aspects such as autonomy and motivation, typical goals of the physical literacy; questions 1 and 2), water competence (in which children’s perception in specific aquatic motor competence and his relationship with the aquatic environment are investigated; questions 3, 4, 5, 6, 7 and 8) and psychosocial aspects related to the performance and relation with peers, (questions 9 and 10). Each response regarded a different level of the perception of the competence (A = high, B = medium; C = low); for the comparison made, positive answers (level A and B) were considered.

#### 2.3.5. Attribution of Colours

**Figure 1:**
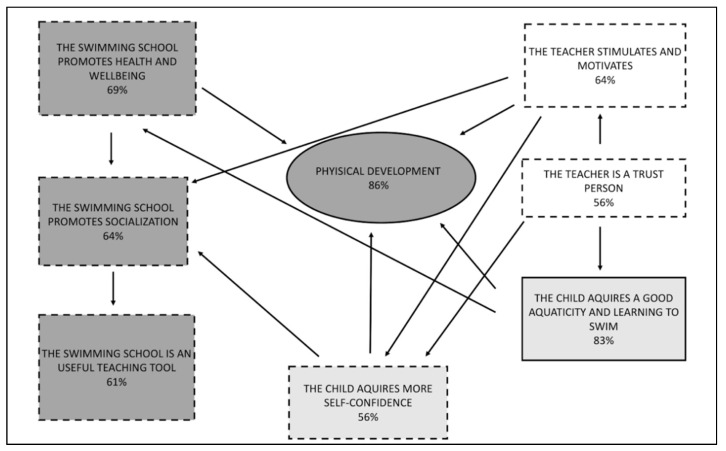
Representation of the themes emerged from the semi-structured interview with parents about considerations related to swimming school and swimming course attended by the children. The core category is represented at the center, in the ellipse with the continuous edge. Rectangles with continuous edges represented the category with the most frequent labels (>75%), those with dashed edges the category with a frequency between 50% and 75%. The dark grey indicates physical literacy’s dimension, the light grey stands for the children’s size, and the white is associated to the instructor’s sphere. The categories with a less frequent labels (<50%) were not considered in the study.

**Figure 2:**
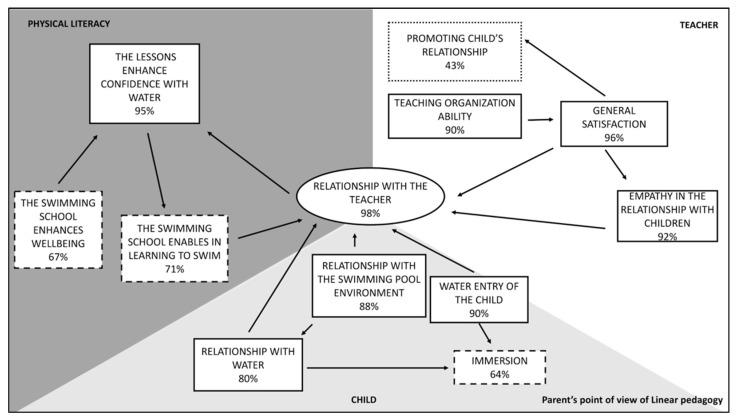
Diagram related to the parents’ perception of LI group as revealed by the administered questionnaire.

**Figure 3:**
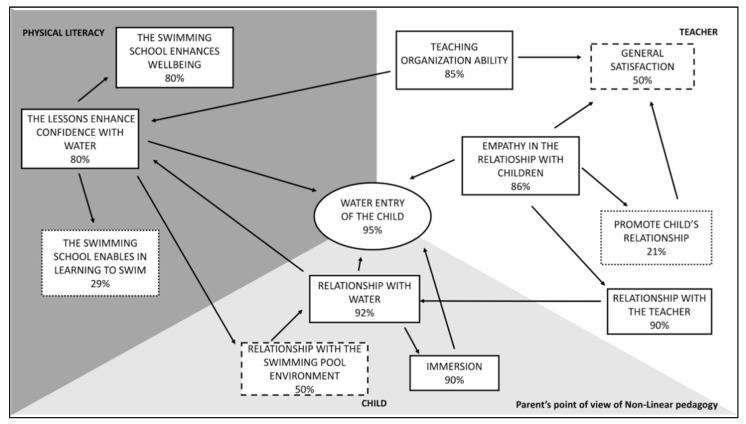
Diagram related to the perception of NL parents’ group according to the results of the questionnaire administered.

**Figure 4:**
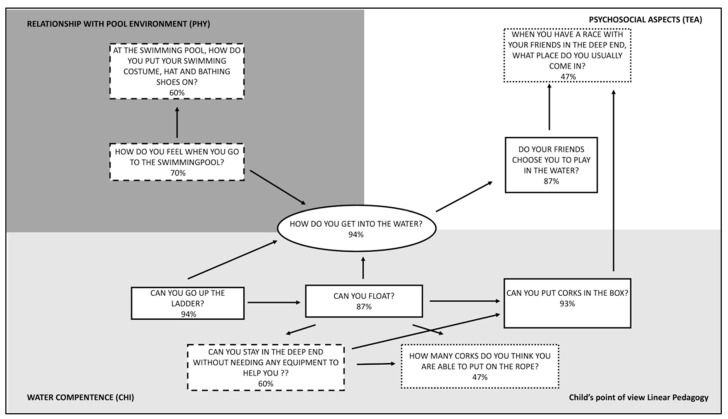
Diagram related to the perception of the children of the LI group according to the results of the pictorial scale indicating the percentage of positive responses.

**Figure 5:**
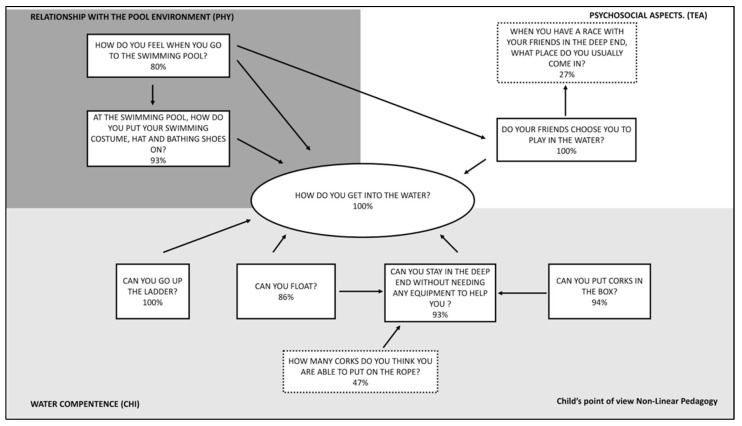
Diagram related to the perception of the children of the NL group according to the results of the pictorial scale indicating the percentage of positive responses.

In order to differentiate the analysis’ approach, in Figures 1–5, we have considered three different colors (arbitrarily chosen) as Grounded Theory method suggests, for a better recognition of categories and thematic areas [24].

In this regard we decided to allocate the dark grey to the answers related to the concept of physical literacy, the light grey to the aspects related to the personal aquatic motor competence of the child, and the white to the answers distinguishing the sphere of the teacher.

### 2.4. Data Analysis

A mixed approach with predominant-qualitative approach for this study was used, in order to combine and integrate the physical aspects, such as aquatic motor competence, and social and psychological aspects, such as parents’ and children’s perceptions.

#### 2.4.1. Qualitative Analysis

A qualitative analysis approach for the results of preliminary interview was led, according to Grounded Theory methodology. Furthermore, parents’ questionnaire and children’s perceived motor competence were administrated as explained previously.

#### 2.4.2. Quantitative Analysis

To analyze the pre-post intervention changes measured with Langerdofer-Bruya test, the non-parametric Wilcoxon test was used, while the Mann-Whitney test was used for delta comparison. For all performed analyses, was considered an α = 0.05. Then the Effect Sizes (ES) were calculated using Phi (φ) and r^2^. Coefficient of variation (expressed as a percentage CV% = (Standard Deviation/Mean) × 100) was calculated in order to identify the intra and intergroup homogeneity.

## 3. Results Qualitative and Quantitative Analysis

### 3.1. Preliminary Semi Structured Interview Proposed to the Parents, Qualitative Analysis

According to the Grounded Theory Method, results can be represented through schemes, as shown below in Figure 1. The diagram illustrates the themes that emerged from semi-structured interview set up with parents, starting from the research question.

Analysis shows that **physical development** resulted in one of the most important reasons that led parents to choose swimming courses **(86%)**. The idea often is enforced by the pediatrician: *“The doctor advised us swimming during the growing phase because (it) is the best sport”* (interview n.12), *“Since my son has flat feet and is a little bit overweight, the pediatrician advised me to take him to the swimming pool”* (interview n.13).

Parents considered the swimming course as a proper teaching tool because the discipline required into the aquatic environment can be conveyed through the game: *“In my opinion swimming can be taught with a playful key, even though concentration is required”*.

The category **acquired good water skill and learning to swim (83%)** represents the main motivation for parents to enroll their children in the pool in order to acquire a good swimming technique: *“I would like her to learn swimming very well, to become a good swimmer, which I’m not.”* (interview n.34), *“We started early because it’s difficult to learn, you must have a precise technique for breathing and coordination of the movements”* (interview n.63). Those who acquired familiarity with water, wanted their children to follow their same example: *“I wanted her to have a beautiful aquaticity since she was a child, to love water like I do.”* (interview n.2), *“I decided for swimming because his father is very sporty, so they can swim together”* (interview n.29).

A state of well-being and health contributes to guaranteeing a child’s physical development regarding the physical literacy dimension, while acquiring more self-confidence constitutes the base to guarantee a good physical development of the child. Physical development is also influenced by the instructor who must be able to stimulate and motivate the child. *“I hope my daughter always grows up with people which could passionate her to sport”* (interview n.35), *“The instructor who gratifies my daughter is a good thing”* (interview n.7).

The relationship based on trust between instructor and child is decisive for the acquisition of a good aquaticity and consequently also for the physical development which happens through aquatic environment: *“The child confidence in instructor is crucial”* (interview n.12), *“the parents in more comfortable if he sees a good teacher”* (interview n.3).

The swimming school is considered as a means to improve and wide the possibility of promoting the socialization and relationship with peers. Sometimes this need is encouraged by the parents: *“I would like my son, through the swimming course, learning to approach with the others feeling at ease”* (interview n. 24), *“Learning to stay in a group and find new friends”* (interview n.20) overall for the lonely and shiny children who have difficulties with social relationships: *“My son is a bit lonely so they recommended us an activity with other children”* (interview n.93) and sometimes this need is searched by the children themselves, who joined the swimming school because of the attendance of their friends: *“He decided himself, probably because of his two school mates who already attended the swimming course, so he wanted to attend it too”* (interview n.70).

### 3.2. Quantitative Analysis Related to Actual Aquatic Motor Competence Tests of the Children

Pre- and post- intra-group results of the linear group (LI) and non-linear group (NL) and inter-group (LI vs. NL) results are shown in Table 2.

The pre- and post- comparison of LI and NL group showed that children improved overall. A more detailed analysis of the LI group showed that children improved all the skills investigated except for water entry, for which no statistically significant differences were emerged.

Within the NL group, appreciable improvement were encountered in competence related to buoyancy, arm propulsion action, and arm recovery action. The comparison between LI and NL showed that, the LI group reached greater progresses than the NL group, particularly in terms of buoyancy, arm propulsion action, and combined movement. NL group, on the other hand, reached greater progress in arm recovery action and in the homogeneity intragroup evaluated with CV%. Considering this last result, NL group has achieved a greater homogeneity at the end of the swimming school (CV% = 12.2%) than the LI group (CV% = 17.9%). In particular for “Breathe control and immersion” (CV% from 18.3% to 12.5%), “buoyancy” (CV% from 39.8% to 21.7%) and “leg propulsion action” (CV% from 40.8% to 21.7%) the NL group increased the homogeneity, which is an indicator of an effective learning. LI group increased the non-homogeneity in all the items except in “water entry” (CV% from 14.3% to 9%) and in “buoyancy” (CV% from 34.5% to 20.6%). 

### 3.3. Global Results Emerged at the End of the Swimming Course in LI e NL Pedagogy Group Related to the Expectations of the Parents and the Perception of the Children

Results emerged from GT are shown in Table 3. Data are presented as percentages.

### 3.4. Specific Results Related to the Parents’ Perception Questionnaire Post Intervention, Qualitative Analysis

The results of the parents’ questionnaires are showed below. Figure 2 presents the diagram according to Grounded Theory that illustrates the results of the questionnaire which the parents of LI group children were subjected to.

**Relationship with the teacher** represents the **Core Category (98%). General satisfaction (96%)** seems strongly dependent on it and it is supported by a good teaching organization.

The empathy created by the teacher with the children allows the development of the relationship with teacher.

The influence of the teacher on the social sphere that includes children and his or her relationship with the peers, is not perceived as fundamental.

The water competences in the child sphere are all related to the Core Category. In fact, in parents’ opinion, a good relationship with the teacher let a confident entry into the water of the child, developing a good relationship with the swimming-pool environment and a good relationship with the water. The **immersion** is a positive experience only for the **64%** of the children.

**Learning to swim** is a more crucial aspect for the parents of the LI group. Positive answers to the question “Do you think that the swimming course has enabled your son /daughter to learn the swimming technique?” account for **71%**. From the parents’ point of view, **well-being** created by the swimming school is crucial for the growth of the children **(67%).**

As shown in Figure 3, the main theme, emerged from the questionnaire vaulted to investigate parents’ NL perception, is related to the **water entry of the child (95%),** which represents the **Core Category**. Water entry requires to have confidence with the water, which is an essential aspect of swim learning. On the other hand, in Figure 3, **learning to swim** does not result to be a relevant aspect in the physical literacy sphere **(29%)**, but rather the **well-being (80%)** of the child. As previously mentioned, getting into the water presupposes that the child is confident and has a good relationship with the water. The positive answers to the question “Does your child feel at ease when he/she/is in water?” in fact are related to the balance, to move into the fluid with the proper pushes and to the mastery of breathing [10]. These are crucial aspects required in order to learn the **immersion,** which represents another obvious theme for the parents of NL group with the **90%** of positive answers.

According to the parents’ point of view, having empathy with the children is important for the teacher in order to facilitate the water entry of the child. On the other hand, would not seem as crucial the ability of the teacher to promote the relationship between the peers.

Teaching organization allows to develop water confidence on the side of the child and indirectly influences the predisposition to entering the water.

**General satisfaction** of the child for parents of children in NL seems to be low **(50%).**

Establishing a good relationship with the swimming pool environment is perceived as a non-strict need in order to promote a relationship with the water and water confidence in an aquatic environment.

### 3.5. Specific Results Related to the Perception of the Aquatic Competence of the Children Post Intervention, Qualitative Analysis

In Figure 4, according to GT methodology, the analysis emerged from the pictorial scale to which the children were subjected is shown.

Most of the children of the LI group **got into the water** happily **(94%)** by jumping into it or entering gradually. Getting into the water with confidence presupposes having a good relationship with it, as well as to be able to go up the ladder in order to exit both in high or shallow water and to float alone or with the help of corks. A good buoyancy allows the children to move easily into the fluid and to reach in, putting corks in a box quite easily both in high and low water.

The perception of a good buoyancy let the child to verify if he or she is able to stay in water without the help of corks, even if it turns out to be more difficult to put corks on the rope. Although there is good buoyancy and displacement in water, this unsureness could be motivated by an alteration of the spatial perception and to the difficulty in controlling the fine coordination in the aquatic environment, as well as to the pushing action of the legs in order to maintain buoyancy [8,10].

Getting into the water with confidence let the children feel at ease in an aquatic environment and promoted a **positive relationship with the swimming pool environment (70%).** The pool environment predisposes them to obtain a greater **autonomy on practical aspects** aimed at personal training such as putting on the swimming costume, the hat, and bathing shoes. This aspect is not completely achieved by all the children yet **(60%)**. A comfortable environment let a better development of their own autonomy and self-efficacy [6]. Getting into the water with sureness grants an image and higher self-perception visible to the other children that predisposes them **to be chosen by their peers to play in the water (87%)**. In fact, not all the children are chosen for play in the water by the others.

All the children of NL group (Figure 5) **get into the water** with ease **(100%)** by jumping into it or entering into it gradually, and all of them managed to exit using the ladder both in high and shallow water. Most of them succeed in floating with and without the help of corks and this allows them to stay in water without invalidating difficulties and to put corks in the box both in high water and in low water. Putting corks on the rope is a quite difficult exercise for the NL group too. From a relationship point of view, the teacher’s ability to create situations encourages the learning and allows the children to develop a sufficient self-esteem in order to promote the relationships within the group. This aspect allows all the children **to be chosen by the peers to play** in the aquatic environment **(100%).** The preparation of the activity by the teacher, according to a non-linear learning approach, let the children develop a greater autonomy, also in the training of the activity as putting the swimming costume, the hat and the bathing shoes by themselves. We can assume that they show **autonomy on practical aspects (93%)**. In fact, all of them are able to use the changing rooms independently. The self-efficacy sense allows them to feel at ease in the **swimming pool environment (80%)**.

## 4. Discussion

### 4.1. Parents’ Preliminary Interview and GT Related to Parents’ Perception

**Figure 6:**
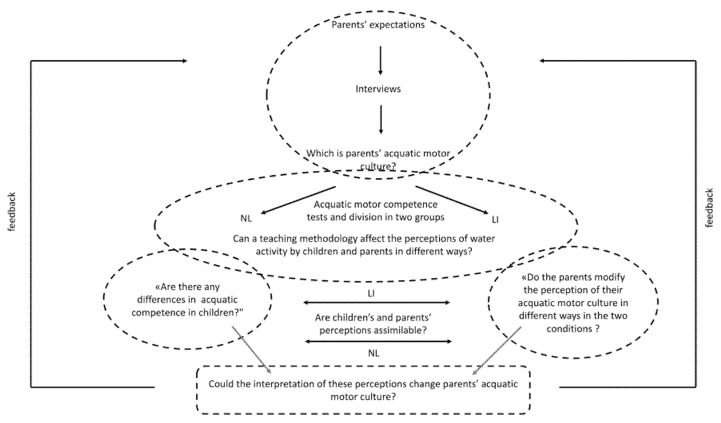
Graphical representation about the fields of investigation of in the research work.

Analysis of the preliminary interview (Figure 6) showed that parents believe aquatic activity has a positive effect on the physical development of the child and that this is also related to the acquisition of good swimming competence and the learning of the technique. The analysis of the interviews also illustrated the importance of improving social and educative aspects. However, the re-processing of the questionnaire’s data at the end of the activity, and after the division into the two groups, reveals two different scenarios.

Comparing parents’ questionnaires of LI and NL groups, 8 out of 12 items presents better results in the LI group. Getting into the water of the child represents the positive core category (95%) of NL group, while “relationship with the teacher” is the main positive aspect in LI group (98%). From this comparison we can learn that the parent of NL group perceives the child at the center of the method, while in the LI group the teacher has a crucial role. As a result of this comparison, a different perception of the swimming school emerges.

In the NL approach great emphasis has given to the creation of a suitable environment in order to allow the development of the competence autonomously, through a three-valued approach as described in the Self Determination Theory (SDT) [25]. In SDT the aspects related to autonomy, competence and social context take shape in the Cognitive Evaluation Theory (CET) mini-theory, in which the intrinsic motivation arises from the satisfaction in achieving results with their own abilities. In CET the perception of competence and the autonomy have a critical role in order to maintain intrinsic motivation, crucial in sport for children’s participation in healthy behaviors across the life span [25,26,27].

The NL group better perceived a positive relationship with water (92%) and immersion (90%) differently from LI group (80% and 64% respectively). The distinction in the perception of these two aspects can be due to the free exploration of the child who discover the environment respecting her/his own learning time, without forcing or anticipating the timing. Immersion is not lived as a constriction, so this method allows the children to a learn this fundamental in a less aggressive way [21].

However, the percentage of positive responses to the question “Do the lessons proposed allowed your son/daughter to have a greater confidence with water and swimming pool environment?” resulted to be higher in LI group (95%) than in the NL group (80%). The percentage of positive responses to the question “Do you think that the swimming course has enabled your son /daughter to learn the swimming technique”, is higher in the LI group (71%) than in the NL group (29%). The LI approach was oriented towards the motor learning, in which the acquisition of swimming technique resulted to be crucial through a systematic and gradual didactic progression. The role of the teacher in LI method was crucial because resulted to be a mediator of a technical learning in a more incisive and determinant way, through the organization of more dynamic and progressive lessons (90% vs. 85%). The level of general satisfaction investigated with the question “Can you be satisfied about the swimming school offered by the instructor?”, resulted to be lower in NL parents’ group than the LI group (50% and 96% respectively): the feedback regarding the immediate learning of swimming technique was related to the aquatic culture of the parents that in the preliminary interview confirmed to be source of satisfaction. These results presuppose the parents’ awareness regarding time and methodology necessary to acquire the fundamental motor skill to reach a proficiency barrier, useful for health and aquatic well-being [28]. Contrarily, this competence needs acquisition of specific knowledge that is not owned by the parents [29]. The impression that the parents had of LI group was that the lesson was organized in a more dynamic and challenging way, and that contributed to a better physical development because turned out to be clearer and more understandable by the observer. This methodology enforced the parent’s idea expressed in the preliminary interview and of the water activity. In LI methodology, social aspects were developed in a different situation than in the NL group because the different organization of the water activity which was obtained with a teaching styles based on reciprocal observations [30]. In order to meet the priority of the parents, the child had to obtain good aquatic competences to ensure a safe future that prevents the risk of drowning [31,32], In the literature is evident the relation between the parent’s involvement and the child’s behavior [33]. It is known that, some variables such as parental relationship may influence the well-being of the child [34]. In particular, aquatic experience of the child based on teacher centered pedagogy (LI), positively evaluated by the parents, could slow down the natural and optimal learning process [35]. This became particularly relevant when the teaching method presented excessive pressure to the learner [36].

### 4.2. Actual Motor Competence and Perceived Motor Competence of the Children

The children of LI group achieved a greater aquatic competence level than the NL group, according to the parents’ expectations. According to literature, the method of how someone learns to swim is significant [37]. As shown in Table 2, the children of the LI group were better by about 7.3% in total results than children of the NL group. NL group instead achieved a greater homogeneity at the end of the swimming school than the LI group. We can assert that non-linear pedagogy, based on student centered pedagogy, contributed to reducing the intra-groups’ competence gap. Data seems to agree to the aims of physical literacy: in the early to middle childhood period is particularly important to consider the individual learning path [36].

Regarding the perceived motor competence of the children, 7 out of 10 items presented better results in NL group. Children of LI and NL group are both able to achieve tasks such as putting on the swimming costume, the hat and the bathing shoes. The NL group perceived a greater positive mood (93% NL vs. 70% LI) and show off a greater autonomy in the changing rooms (“At the swimming pool, how do you put your swimming costume, hat and bathing shoes on?” 60% LI and 80% NL). The guided discovery and the free exploration of non-linear pedagogy let the child develop a greater self-efficacy because the child is allowed to reach the steps of the learning path within his/her own time [36]. This freedom allows him/her to acquire more confidence in his/her own competences, developing a greater autonomy even in tasks outside the swimming pool. The literature reported that the greater the perception of aquatic competence experienced by the participants, the greater their real aquatic competence. The teacher would be responsible for reinforcing a positive self-concept in children from three to six-years-old, to promote the development of perceived aquatic motor competence and help them to overcome fears which could compromise the development of certain actions [38,39]. 47% of the children in LI group and 27% of the children in NL group claim to be among the first places during races. We can assume that a student-centered pedagogy approach, as in NL group, didn’t emphasis the competition in the activity proposed, while the opposite occurred in teacher-centered pedagogy, as in LI group (“When you have a race with your friends in the deep end, what place do you usually come in?” 47% LI and 27% NL).

This may depend on the emphasis given by the teacher (more oriented in a theory based on performance and less on the task), on the comparison with the peers, and on how much the teacher stimulated the aspects related to the competition. Furthermore, learning become difficult when the activity is compulsory and does not always respect the learning time of the individual [40,41].

Both the children of LI group (87%) and the children of NL group (100%) affirm to be frequently chosen by the peers during games (“Do your friends choose you to play in the water?”). Relationship with the peers resulted to be well structured both in LI and in NL group since the dynamics, given by a greater autonomy derived from a greater self-esteem, were directly managed by the peers, with a limited intervention of the teacher. In LI group, the direct approach of the teacher may have influenced some dynamics [36].

Respecting the timing of achieving the aquatic competences related to stay in water without the help of corks in NL group (93%) allowed the child to perceive better his/her self-efficacy than in the LI group (60%). Developing a perceived motor competence, based on own actual capacity, allows a wellbeing status indispensable to build a physical literacy that includes affective, physical, cognitive and behavioral spheres [1].

### 4.3. Practical Applications

The aquatic motor culture of the parents can be modified through increasing their awareness about the differences and gaps between their perception and the children’s perception. Moreover, the evaluation of the homogeneity of the group could be a useful tool in order to verify the effectiveness of the learning. In particular, the increasing of homogeneity in the NL group, as evidenced by the results related to the actual motor competence tests, can be recognized as an index of effectiveness of the learning. In the LI group, the decreasing of homogeneity in the results of the same tests, allowed the children to improve their aquatic technique evidencing the gap between the ones which are more able than others. This gap demotivates the most vulnerable children. This educational view of the swimming school could be motivated during the preliminary meeting with the parents.

We were able to see that the consideration that parents often have initially regarding the motor activity is strictly linked to the performance and the achievement of results; it is difficult for them to consider the psycho-physical and social well-being of their children. Relating the parents to the point of view of the children could enable them to modify their knowledge related to the aquatic motor culture, also taking into account the relational, emotional and psychological aspects, thus helping to raise independent and self-confident children. For this reason, the swimming school should have a more formative and educational vision, with the aim to lead the parent towards the awareness that the children and their needs must be placed at the center of learning in order to let them live a positive life with positive effects on their physical literacy and health [27].

## 5. Conclusions

In conclusion, both children and parents’ group presented substantial differences related to the perception of the activity proposed.

We can affirm that NL approach was more appreciated by the children than the LI approach, while for the parents’ group, LI method resulted to be more rewarding than the other because the initial expectation were satisfied. A preliminary interview let us verify the children’s behaviors expected by the parents at the end of the course. It was a useful tool to expand the parents’ knowledge related to their aquatic motor culture, highlighting the aspects related to autonomy, self-efficacy, achieved self-esteem and achievement of a healthy status in anatomical, physiological, psychological and sociological terms that can be conveyed by the swimming activity. We retained interesting to note that children obtained more evident progress in learning the swimming technique through the LI method, but there was a gap in what they perceived and what they achieved through the motor tests [42]. On the other hand, the children who learned with the NL method, developed an higher sense of awareness, self-efficacy (in changing rooms and in the relationship with the water) and self-esteem (in the relationship with the peers), which implies a greater assertion of soft skills [28]. The gap between the perceived competence and the actual competence depends on the lack of consciousness of their own abilities. This rare awareness is crucial in children which are between 4 and 8-years-old and this is due to the inability to abstract thoughts. With the growth and development of logical thinking, the child will be able to reduce the gap between perceived and actual motor competence [43].

This reflection is crucial for the didactic planning of the motor development of the child. It would be appropriate to emphasize how the educational view, differently from the utilitarian and sportive approach, could stimulate, referring to the physical literacy culture which include the individual need [44], the solidarity and attention to the most vulnerable children, improving their self-esteem through a better perception of motor competence.

## Figures and Tables

**Table 1:** Comparison of the main methodological principles regarding teacher-centered (LI) and student-centered (NL) pedagogies.

Methodological Principles	
**Teacher-Centered Pedagogy** [20] **Linear Group**	**Student-Centered Pedagogy** [21] **Non-Linear Group**
Specific training aimed to immersion, buoyancy and propulsion	No specific training aimed to immersion, buoyancy and propulsion
Proposals due to a physical effort of the child (muscular, cardio-respiratory)	Discovery of the aquatic environment for a own initiative and not as a result of a external imposition
Proposals aimed to promote a rapid autonomy and independence in water	Ludic interaction between children and between children and teacher
The instructor stimulates and proposes specific exercises aimed at a gradual and progressive conquest of the aquatic environment	Respect for the child, for his/her emotional reaction and learning rhythms
Use of the metaphors and imitative exercises. Gradual progression of exercises aimed at the learning of a specific operative goal at a time	Teaching aids and materials as stimuli of learning. Randomized a varied multilateral approach aimed at testing multiple motor possibilities simultaneously

**Table 2:** Results of Pre and Post Langerdofer-Bruya aquatic motor competence tests, expressed as mean ± standard deviation. Statistical differences (*p* < 0.05) of intra-group analysis are reported in Pre vs. Post columns. Statistical differences (*p* < 0.05) of inter-group analysis are reported “LI vs. NL pre” and “LI vs. NL Δ” columns. ES = effect size. * *p* < 0.05.

	Linear			Non-Linear			Linear		Non-Linear		LI vs. NL Pre	ES (φ)	LI vs. NL Δ	ES (φ)
	Pre (A.U.)	Post (A.U.)	Δ (A.U.)	Pre (A.U.)	Post (A.U.)	Δ (A.U.)	Pre vs Post	ES (r^2^)	Pre vs Post	ES (r^2^)				
Water entry	2.8 ± 0.4	2.9 ± 0.3	0.1 ± 0.4	3.0 ± 0.2	3.0 ± 0.1	0.0 ± 0.3	0.059	0.040	0.143	0.004	0.023 *	0.058	0.143	0.024
Breathe control and immersion	2.8 ± 0.4	3.2 ± 0.5	0.5 ± 0.7	2.5 ± 0.5	2.9 ± 0.4	0.2 ± 0.5	0.001 *	0.131	0.138	0.080	0.166	0.022	0.138	0.025
Buoyancy	1.6 ± 0.5	4.0 ± 0.8	2.5 ± 1.0	1.7 ± 0.7	3.4 ± 0.7	1.7 ± 0.9	0.000 *	0.357	0.001 *	0.380	0.674	0.002	0.001 *	0.129
Arm propulsion action	1.0 ± 0.0	2.3 ± 0.4	1.3 ± 0.4	1.0 ± 0.0	2.0 ± 0.0	1.0 ± 0.0	0.000 *	0.388	0.000 *	0.539	1.000	0.000	0.000 *	0.163
Arm recovery action	1.0 ± 0.0	2.3 ± 0.5	1.3 ± 0.5	1.0 ± 0.0	2.5 ± 0.5	1.5 ± 0.5	0.000 *	0.385	0.048 *	0.435	1.000	0.000	0.048 *	0.044
Leg action	1.0 ± 0.0	3.2 ± 0.5	2.2 ± 0.5	1.1 ± 0.4	3.1 ± 0.7	2.0 ± 0.7	0.000 *	0.381	0.146	0.422	0.355	0.010	0.146	0.024
Combined movement	1.0 ± 0.0	2.5 ± 0.6	1.5 ± 0.6	1.0 ± 0.0	2.4 ± 0.5	1.4 ± 0.5	0.000 *	0.369	0.298	0.439	1.000	0.000	0.248	0.012
Total	11.2 ± 1.4	20.5 ± 3.7	9.4 ± 2.6	11.3 ± 1.1	19.1 ± 1.3	7.8 ± 1.4	0.000 *	0.352	0.008 *	0.414	0.725	0.001	0.008 *	0.08

**Table 3:** The Table is divided into three thematic areas. On parents’ perception side, the first line refers to the physical literacy’s sphere, the second line is related to the child dimension and the last one regards with the aspects of the instructor. On children’s perception side, the first line refers to the relationship with the pool environment, the second one is related to the water competence and the last one regards with the psychosocial aspects. The three categories of parents’ perception emerged from the results of GT, of which the questionnaire has been structured on. The three categories of children’s perception have arbitrarily made to correspond to the emerging GT categories. The percentages considered refer to positive responses.

Percentage Data Are Shown below (Table 3). Education							
Parent’s Perception				Child’s Perception			
		**LI**	**NL**			**LI**	**NL**
**Physical literacy** **(PHY)**	1.1. Do you think that the swimming school has been beneficial for the health, the physical development and the wellness of your son/daughter? A. Yes, completely B. Yes, partially C. Not at all D. No, I don’t.	67%	80%	**Relationship with pool environment** **(PHY)**	1. At the swimming pool, how do you put your swimming costume, hat and bathing shoes on? A. I do it myself B. Sometimes I do it myself C. My teacher always helps me	60%	80%
	1.2. Do you think that the swimming course has enabled your son /daughter to learn the swimming technique? A. Yes, completely B. Yes, partially C. Not at all D. No, I don’t.	71%	29%		2. How do you feel when you go to the swimming-pool? A. I love going to the pool B. It’s hard to go to the pool C. They make me go to the pool	70%	93%
	1.3. Do the lessons proposed allowed your son/daughter to have a greater confidence with water and swimming pool environment? A. Yes, completely B. Yes, partially C. Not at all D. No, they don’t.	95%	80%				
**Child** **(CHI)**	2.1. How does your son /daughter relate in the swimming pool environment? A. He/she is confident B. He/she feels at ease C. He/she needs support D. He/she doesn’t want to go to the swimming pool	88%	50%	**Water competence** **(CHI)**	3. How do you get into the water? A. I jump in right away B. I get into the water gradually C. I don’t want to get into the water	94%	100%
	2.2. How does your son/daughter get into the water? A. He/she jumps in right away B. He/she get into the water gradually C. He/she needs support D. He/she doesn’t want to get into the water	90%	95%		4. Can you go up the ladder? A. I can get up it in the deep end B. I can only get up it in the shallow end C. I can’t get up the ladder	94%	100%
	2.3. How does he/she live the immersion? A. He/she enjoys it a lot B. He/she feels at ease during the immersion C. He/she doesn’t like it D. He/she is scared about it	64%	90%		5. Can you float? A. I can float on my own B. I can float with the help of floats C. I can’t float	87%	86%
	2.4. Does your child feel at ease when he/she is in water? A. Always B. Usually C. Sometimes D. Never	80%	92%		6. Can you stay in the deep end without needing any equipment to help you? A. Yes, I can do that B. Only sometimes C. No, I can’t do that	60%	93%
					7. Can you put corks in the box? A. Yes, a lot and in the deep end B. Yes, but only in the shallow end C. I can’t put any in	93%	94%
					8. How many corks do you think you are able to put on the rope? A. A lot in the deep end B. A lot in the shallow end C. None	47%	47%
**Teacher** **(TEA)**	3.1. How is the relationship between your child and the teacher? A. Very positive B. Positive C. Negative D. Very Negative	98%	90%	**Psychosocial aspects** **(TEA)**	9. When you have a race with your friends in the deep end, what place do you usually come in? A. One of the first B. In the middle C. One of the last	47%	27%
	3.2. Can you be satisfied about the swimming school offered by the teacher? A. Yes, completely B. Yes, partially C. Not at all D. No, I can’t.	96%	50%		10. Do your friends choose you to play in the water? A. They always ask me to play B. Sometimes they ask me to play C. They never let me play	87%	100%
	3.3. Has the teacher been able to create an empathic relationship with the children? A. Yes, completely B. Yes, partially C. Not at all D. No, he hasn’t.	92%	86%				
	3.4. Do you think that the teaching organization has been effective? A. Always B. Usually C. Sometimes D. Never	90%	85%				
	3.5. Do you think that the teacher has promoted the relationship between peers? A. Yes, completely B. Yes, partially C. Not at all D. No, he doesn’t.	43%	21%				

## Data Availability

The data presented in this study are available on request from the corresponding author. The data are not publicly available due to a privacy policy.
